# Feature Review Papers in Stroke and Cerebrovascular Disease

**DOI:** 10.3390/jcdd12080309

**Published:** 2025-08-15

**Authors:** Narayanaswamy Venketasubramanian

**Affiliations:** Raffles Neuroscience Center, Raffles Hospital, Singapore 188770, Singapore; ramani_nv@rafflesmedical.com; Tel.: +65-6311-1111

The field of stroke is quickly evolving, with nearly half a million publications in Pubmed alone [1]. It is difficult for clinicians to be updated on every aspect of this disease that has a wide impact on individuals, families, societies and healthcare systems. This Special Issue on Feature Review Papers in Stroke and Cerebrovascular Disease seeks to crystalise key information on selected topics that would be of interest to clinicians, epidemiologists and health authorities. The papers in this Special Issue focus on the challenges in providing stroke treatment in Bangladesh, new information on emerging stroke risk factors, an update on left atrial appendage exclusion, and the potential role of xanthine oxidase inhibitors for stroke.

Stroke is a major cause of mortality and morbidity. Based on the Global Burden of Disease study, in 2021, strokes led to 7.3 million (95% UI 6·6–7·8) deaths, 11.9 million (95% UI 10.7–13.2) incident cases, 93.8 million (95% UI 89.0–99.3) prevalent cases, and 160.5 million (95% UI 147.8–171.6] Disability Adjusted Life Years Lost (DALYs) from premature death or living with disability [2]. Stroke epidemiology is generally well-described in Western Europe and North America; however, data from Asia, especially for lower income countries, are lacking [3], with accompanying challenges in providing adequate stroke care. In this Special Issue, Aziz et al. (contributor 1) report the results of a timely narrative review of epidemiology, risk factors and acute stroke services in Bangladesh, a low–middle income country. Their systematic search of publications in four databases revealed a stroke prevalence of 3 per thousand persons, with geographical variations and a higher rate in males, especially rural males compared to urban males. Compared to Western populations, a higher frequency of haemorrhagic stroke, cerebral venous thrombosis, and intracranial atherosclerosis as compared to extracranial atherosclerosis was found. Novel risk factors included squatting, arsenic in drinking water, betel-nut chewing, chewing (smokeless) tobacco, and low serum folate. There was a high rate of stroke recurrence in the paediatric population that was attributed to intracranial vasculopathy, particularly vascular narrowing and moyamoya disease. Acute stroke services were found only in tertiary-level hospitals or a few private hospitals. There were no stroke units, and treatment was administered by either a general practitioner or a neurologist. Thrombolysis was very limited due to the high cost of tPA, late patient presentation due to a lack of knowledge about stroke, and a lack of an infrastructure facilitating early arrival of acute stroke patients; thrombectomy had very low availability. Rehabilitation services were not easily accessible, though two non-governmental organisations provided long-term stroke rehabilitation services together with primary prevention for those who were unable to afford treatment. These issues are frequently seen in lower and lower–middle income countries as they struggle with their burden of stroke; workable sustainable solutions are needed [4]. This will need a government-wide approach with the close involvement of private sector healthcare, the pharmaceutical industry and active engagement of the community.

The traditional risk factors for stroke include non-modifiable factors such as increasing age and male sex, and modifiable factors such as hypertension, diabetes mellitus, dyslipidaemia, cigarette smoking, obesity, low physical activity and unhealthy diets [2]. Detection and adequate treatment of these factors are crucial for the primary prevention of stroke [5]. In this Special Issue of *JCDD*, Hameed et al. (contributor 2) review emerging risk factors for stroke, including air pollution and climate change, gut microbiota, high altitude, and systemic infection. Air pollution accounts for 14% of all stroke-associated mortality. Associations have been found with concentrations of coarse (<10 μm) (PM10) and fine (<2.5 μm) (PM2.5) particulate matter and stroke admissions, both ischaemic and haemorrhagic. Both extremely high and low temperatures have been linked to an increase in stroke incidence and mortality; even long-term exposure to temperature variability has been associated with increased stroke incidence. By releasing various metabolites, the gut microbiome is able to impact the central nervous system; alterations in the composition and function of gut microbiota have been associated with infarct volumes and neurological status, paving the way for possible interventions using probiotics, prebiotics, or faecal microbiome transplantation. There are conflicting data on the association between altitude and stroke, with a possible protective effect at high altitudes (1500–2500 m) but detrimental effect at very high altitudes (>3500 m). Infective endocarditis and Chagas’ disease can cause cardioembolic stroke, while intracranial infections such as meningitis by various organisms may led to stroke due to effects on the intracranial arteries. It is becoming increasingly clear that systemic infections, both acute and chronic, increase stroke risk, making it crucial that infections are appropriately treated.

Approximately 17% to 30% of strokes have been attributed to cardioembolism from atrial fibrillation (AF) [6]. In patients with stroke or transient ischaemic attack attributed to nonvalvular AF, oral anticoagulation with warfarin or directing acting oral anticoagulants (DOACs) (e.g., apixaban, dabigatran, edoxaban or rivaroxaban) is recommended to reduce the risk of recurrent stroke [7]. However, in those who have contraindications to lifelong anticoagulation, percutaneous closure of the left atrial appendage (LAA), the source of most of the emboli, may be considered. Various closure devices are commercially available, with different safety profiles and complications. Karpierz et al. (contributor 3) reviewed the anatomy, physiology, and functions of the LAA and available LAA closure devices, reviewing their clinical outcomes, as did Swieczkowski et al. (Contributor 4). The LAA can be surgically excluded or removed or an epicardial device can be applied or non-invasively excluded by the insertion of an endocardial device by a percutaneous trans-catheter approach. Epicardial devices include AtriClip, LARIAT and Seirra, while the endocardial devices include Amlatzer Amulet, LAmbre, WATCHMAN ad WaveCrest. Overall, LLA closure has been shown by meta-analyses to lead to significantly fewer thromboembolic events, improved post-operative mortality rates, and efficacy comparable to DOACs. But each device has its benefits and risks and limitations, and post-procedure management. Thus, the treating physician needs to make a careful selection of a device suitable for that specific patient, assisted by imaging, so as to maximise the benefit and minimise the risk.

Xanthone oxidase (XO) catalyses the conversion of hypoxanthine from purine bases to uric acid, during which reactive oxygen species are produced that may lead to endothelial dysfunction, functional and metabolic impairment, and activation of inflammation [8]. A meta-analysis has shown that hyperuricemia increases stroke incidence and mortality [9]. Another meta-analysis showed that higher serum uric levels were associated with a better outcome after stroke [10], while another analysis showed no association between serum uric levels and stroke prognosis [11]. In view of this conflicting information, and the potential role of XO inhibitors (XOIs) in cerebrovascular disease, Bai et al. performed a systematic review and meta-analysis of the effect of XOI in the prevention and treatment of stroke (Contributor 5). They analysed 14 studies (*n* = 115,579). XOIs did not significantly reduce the stroke risk, based on randomised controlled trials and cohort studies of allopurinol or febuxostat. However, they were associated with improved functional outcomes after stroke based on the modified Rankin scale score in one randomised controlled trial using allopurinol. Other findings included a decrease in the levels of intercellular adhesion molecule-1 levels, improvement in the augmentation index, reduction central blood pressure, and delayed progression of carotid intima-media thickness. These results suggest that XOI should not be used to prevent stroke. More multi-centre randomised double-blind placebo-controlled trials are needed before recommending XOI as a potential treatment to aid post-stroke recovery.

The review papers in this Special Issue of the journal have crystalised the current knowledge on stroke epidemiology and care in Bangladesh, emerging stroke risk factors, LAA occlusion and XOI in stroke. This information will be helpful for those seeking up-to-date information on these fast-evolving areas. Clearly, more research is needed.

## Data Availability

The original contributions presented in this study are included in the article. Further inquiries can be directed to the corresponding authors.
